# Zwischen Sinnfindung und Selbstverwirklichung: eine qualitative Studie zur Inanspruchnahme von Hilfe aus Sicht der pflegenden Angehörigen

**DOI:** 10.1007/s00391-025-02437-1

**Published:** 2025-04-08

**Authors:** Jenny Kubitza, Verena Steinmaier, Eckhard Frick

**Affiliations:** Technische Universität München, TUM School of Medicine and Health, Klinik und Poliklinik für Psychosomatische Medizin und Psychotherapie, TUM Klinikum, Technische Universität München, München, Deutschland

**Keywords:** Pflegeperson, Häusliche Pflege, Unterstützung, Spiritualität, Inhaltsanalyse, Informal caregivers, Home care, Support, Spirituality, Content analysis

## Abstract

**Hintergrund:**

Pflegende Angehörige (PA) nutzen kaum formelle und informelle Hilfen, was sich auf ihre Belastung und Lebensqualität auswirken kann. Faktoren, die zur Ablehnung führen, sind häufig psychosozial (Scham, Schuld, Fremdbestimmung, soziale Normen). Bisherige Studien zu psychosozialen Gründen haben die spirituelle Dimension vernachlässigt. Das Ziel dieser Studie ist es, psychosoziospirituelle Gründe für die (Nicht‑)Nutzung von Hilfsangeboten von PA zu erfahren.

**Methodik:**

Im Rahmen der qualitativen Studie wurden zwischen August 2022 und Juli 2023 leitfadengestützte Interviews mit 24 PA geführt und inhaltsanalytisch nach Mayring ausgewertet.

**Ergebnisse:**

Formelle und informelle Hilfe wird von PA beansprucht, um eine Ausgewogenheit zwischen den eigenen Bedürfnissen und denen der gepflegten Personen zu finden, interpersonale Grenzen zu entwickeln und zu wahren. Hilfe wird aufgrund von schlechten Erfahrungen mit Hilfsangeboten sowie eigenen, familiären und gesellschaftlichen Rollenerwartungen abgelehnt. Solange die Pflegetätigkeit als alleiniger Lebenssinn verstanden wird, kann Hilfe von außen als bedrohliche Infragestellung wahrgenommen werden.

**Schlussfolgerung:**

PA nehmen Hilfsangebote in Anspruch, wenn diese ihre Lebenssituation berücksichtigen und mit den eigenen Werten und Zielen vereinbar sind. Eine psychosoziospirituelle Beratung sollte vor einer Leistungsberatung stehen.

**Graphic abstract:**

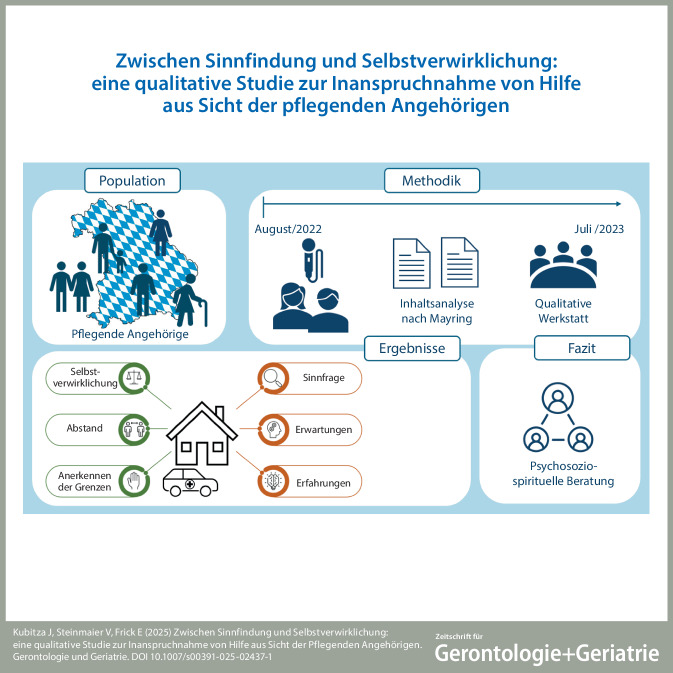

**Zusatzmaterial online:**

Zusätzliche Informationen sind in der Online-Version dieses Artikels (10.1007/s00391-025-02437-1) enthalten.

## Abstract (en)

**Background:**

Family caregivers (FC) rarely utilize formal and informal help, which affects their burden and quality of life. Factors that result in rejection are often psychosocial (shame, guilt, heteronomy and social expectations). Previous studies on psychosocial reasons neglected the spiritual dimension. This study aims to identify the psychosociospiritual reasons for the (non)utílization of help from the perspective of FCs.

**Methods:**

This qualitative study conducted semi-structured interviews with 24 FCs between August 2022 and July 2023 and analyzed according to Mayring.

**Results:**

The FCs request formal and informal help to find a balance between their needs and those of the persons in need of care and to develop and maintain interpersonal boundaries. Help is rejected due to negative experiences with help offers as well as personal, familiar, and social role expectations. As long as nursing care is seen as the only meaning in life, help from outside can be perceived as a threat.

**Conclusion:**

When utilizing help, it is not only about the quality of the offered help but also about its compatibility with oneʼs own values and aims. Psychosociospiritual counselling should be provided before counselling for benefits.

In Deutschland werden 4,9 Millionen (Mio.) pflegebedürftige Menschen zu Hause versorgt, davon werden 3,1 Mio. vorwiegend durch Angehörige (PA) gepflegt [1]. Um die häusliche Pflege zu stärken, können PA auf Unterstützungsangebote zurückgreifen. Das schließt formelle (professionelle), zumeist vergütete Hilfen (z. B. Pflegedienst, Tagespflege) und informelle, in der Regel unbezahlte Hilfen (z. B. Freunde, Nachbarschaft) ein [2].

In der VDK-Pflegestudie wurden deutschlandweit 53.678 PA gefragt, welche Hilfen sie beanspruchen: Von den befragten PA nutzen 38 % den Pflegedienst, 31 % die Verhinderungspflege, 14 % die Kurzzeitpflege, 7 % die Tages‑/Nachtpflege und 7 % gar keine formelle Hilfe. Damit verzichtet die Mehrheit der befragten PA auf formelle Hilfe. Auf informelle Hilfe greifen 50 % der PA zurück [3]. Die EUROFAMCARE-Studie hat hierzu 1003 PA in Deutschland befragt und festgestellt, dass die Familie im Durchschnitt einmal im Monat hilft [4].

PA lehnen Hilfe ab, weil sie zu Angeboten und deren Finanzierung kaum etwas wissen [3, 5], die Angebote nicht verfügbar oder finanzierbar [3, 5] sowie wenig flexibel und bedürfnisorientiert sind [2, 3, 5]. PA nehmen Hilfe als Einschränkung ihrer Partizipation und Unabhängigkeit wahr [3, 5]; das entspricht selten ihrem Anspruch einer guten Pflege [3–5]. Traditionelle Rollenbilder und Meinungen des sozialen Umfelds führen dazu, dass PA sich schämen und schuldig fühlen, wenn sie um Unterstützung bitten [6]. Ob PA Hilfe akzeptieren können, hängt von deren psychosozialen Faktoren (Wissen, Einstellung, Kontrolle, soziale Norm) ab [4, 5].

Die psychosozialen Faktoren umfassen psychische und soziale Bedingungen, die sich gegenseitig beeinflussen. PA werden im Kontext der umgebenen Umwelt betrachtet [7]. Dabei werden psychosoziale Faktoren bisher von den spirituellen abgegrenzt. Spiritualität ist jedoch in die Lebenswelt von PA eingebettet und stellt eine wichtige Bewältigungsstrategie für sie dar [8, 9].

Spiritualität umfasst die Suche und das Erfahren von Sinn sowie existenzielle Fragen [10]. Puchalski und Ferrell beschreiben Spiritualität als Teil des Menschen, der nach Heilung und Versöhnung mit sich selbst oder anderen strebt [11]. Damit hilft Spiritualität insbesondere im Umgang mit Herausforderungen des Lebens wie z. B. im Angesicht von Krankheit, Pflegebedürftigkeit, Krise, Verlust und/oder dem bevorstehenden Tod. Spiritualität trägt zu Selbstreflexion, persönlicher Entwicklung, Freiheit, Freude und Identität bei, gibt ein Gefühl der Verbundenheit mit sich selbst, anderen und/oder einer höheren Geistigkeit und führt zu Halt sowie Orientierung im Leben [10].

Spiritualität unterstützt PA dabei, den Blick nach innen zu richten, und verbessert die Selbstwahrnehmung und das Selbstmitgefühl [12]. Spiritualität bewirkt, dass PA die eigenen Bedürfnisse wahrnehmen, Grenzen formulieren und eine bewusste Beziehung zu sich selbst entwickeln [8, 9]; diese Besinnung auf die eigenen Gefühle trägt zu einer achtsamen Lebenseinstellung bei [12], was ein entscheidender Faktor ist, wenn es darum geht, ob PA Hilfe beanspruchen.

Spiritualität als Bedürfnis von PA sollte daher bei der Untersuchung, warum Hilfe von PA (nicht) beansprucht wird, berücksichtigt werden.

Die Forschungsfrage der Studie lautet:

Welche psychosoziospirituellen Faktoren beeinflussen die Ablehnung bzw. Inanspruchnahme von formellen und informellen Hilfsangeboten bei pflegenden Angehörigen?

## Methodik

### Studiendesign

Die Untersuchung wurde im Rahmen einer Mixed-Methods-Längsschnittstudie zu Bedürfnissen und Bedarfen von PA in Kooperation mit dem Zentrum für Medizinische Versorgungsforschung der Universität Erlangen umgesetzt. Der quantitative Teil untersucht, wie formelle Hilfe sich auf verschiedene Aspekte des Lebens von PA im zeitlichen Verlauf auswirkt, und welche Bedürfnisse PA bezüglich der Hilfe haben. Der qualitative Teil untersucht die spirituellen Bedürfnisse von PA. Dabei wurde deutlich, dass Spiritualität die Inanspruchnahme von Hilfsangeboten beeinflusst. Die Untersuchung wurde angepasst, und in weiteren Interviews wurde nachgefragt, welche psychosoziospirituellen Faktoren auf die (Nicht‑)Inanspruchnahme von Hilfsangeboten wirken. Die Ergebnisse werden in diesem Beitrag vorgestellt.

Die Studie wurde von der Ethik-Kommission der TU München genehmigt (Projekt-ID: 2022-416-S-ND). Sie wird gemäß der „Guideline for qualitative research“ des Equator-Netzwerks vorgestellt [13]. Die vollständige Checkliste befindet sich im Zusatzmaterial online: Anhang.

### Teilnehmende und Rekrutierung

Um möglichst unterschiedliche Erfahrungen im Hinblick auf die Forschungsfrage zu erfassen, wurden PA nach dem Prinzip der maximalen Variationsbreite ausgewählt [14]. Die Teilnehmenden wurden nach vorab definierten Einschlusskriterien bestimmt: 1) ≥ 18 Jahre; 2) übernehmen die Pflege einer Person im häuslichen Setting, wobei sich PA und pflegebedürftige Person keinen Haushalt teilen müssen; und 3) verfügen über ausreichende Deutschkenntnisse.

Für die Rekrutierung wurden Flyer in Selbsthilfegruppen und sozialen Netzwerken verteilt. Zudem wurde mit dem Zentrum für Medizinische Versorgungsforschung und dem Medizinischen Dienst der Krankenkassen (MDK) kooperiert. Der MDK verwies im Rahmen der Begutachtungen auf die Mix-Method-Studie. Sofern die PA der zu begutachtenden Personen an der Studie teilnehmen wollten, leitete der MDK die Adressdaten an die Forschenden vom Zentrum für Medizinische Versorgungsforschung weiter. Diese versendeten den Fragebogen der quantitativen Studie sowie den Flyer der qualitativen Studie postalisch an 404 PA. Sofern Personen an der qualitativen Studie interessiert waren, konnten sie das Team der Professur kontaktieren. Nach mündlicher und schriftlicher Aufklärung wurde von allen Teilnehmenden ein schriftlicher Informed Consent eingeholt. Die Teilnehmenden wurden darüber informiert, dass sie ihre Einwilligung bis zur Anonymisierung der Daten zurückziehen können. Die anonymisierten Daten sind nur für autorisierte Personen zugänglich und werden nach 10 Jahren gelöscht.

### Datensammlung

Zwischen August 2022 und Juli 2023 wurden Interviews mit PA in Bayern geführt (H.K., J.K., V.S.). Die Interviews wurden mit einem Leitfaden vor Ort, per Telefon oder per Videokonferenz durchgeführt. Der Leitfaden wurde nach dem SPSS-Prinzip entwickelt: Sammeln von Fragen, Prüfen der Fragen, inhaltliches Sortieren der verbliebenen Fragen und Subsumieren der Fragen nach Indikatoren zur Beantwortung der Forschungsfrage (E.F., J.K.) [15]. Am Ende bestand der Leitfaden aus 4 Themen: Erfahrungen, Stärken, Bedeutung und Herausforderungen. Dabei wurden die Teilnehmenden zu verschiedenen Aspekten der Pflege befragt und wie diese Aspekte mit ihrer Spiritualität zusammenhängen, z. B. sollten die PA bei ihren Erfahrungen einen Tag beschreiben, aber auch erklären, ob der Pflegealltag ihren inneren Frieden/ihr spirituelles Wohlbefinden beeinflusst.

Kein Thema enthielt explizit Fragen zu Hilfen. Die PA sprachen das Thema von sich aus an. Durch Nachfragen zu Vor- und Nachteilen von Hilfen sowie deren Wirkung auf Wohlbefinden, Einstellung und Rolle als Pflegeperson wurden die PA angeregt, weiterzuerzählen.

### Datenanalyse

Alle Interviews wurden aufgezeichnet und nach Talk in Qualitative Social Research transkribiert (H.K., J.K., V.S.) [16]. Die anonymisierten Transkripte wurden mit MAXQDA (VERBI GmbH, Berlin, Deutschland) Version 22.7.0 inhaltsanalytisch nach Mayring ausgewertet (JK, VS). Ziel der Inhaltsanalyse ist es, die Textmenge zu reduzieren und wesentliche Inhalte als „Abbild des Grundmaterials“ in sog. Kategorien zusammenzufassen [17]. Erste Kategorien wurden deduktiv entlang der Literatur erstellt. Sofern in den Texten neue Themen identifiziert wurden, wurden induktiv neue Kategorien gebildet, wodurch ein Kategoriensystem entstand. Die ersten 3 Transkripte wurden doppelt kodiert, um Eindrücke zu Textpassagen und Themen zu vergleichen. Das entwickelte Kategoriensystem wurde zwei Mal in qualitativen Werkstätten modifiziert (D.T., E.F., J.K., R.M., V.S.).

## Ergebnisse

### Beschreibung der Teilnehmenden

44 PA willigten in die Studienteilnahme ein. Nach 21 Interviews zeigten sich keine neuen Kategorien; drei weitere Interviews wurden organisiert, um das Kategoriensystem zu kontrollieren. Insgesamt wurden 24 Interviews geführt.

Es wurden 14 Frauen und 10 Männer befragt, die Lebenspartner/-innen (*n* = 12), (Schwieger)Eltern (*n* = 10) oder Kinder (*n* = 2) pflegten. Die PA waren durchschnittlich 63 Jahre alt (Min.: 32; Max.: 81) und pflegten im Mittel seit 5,4 Jahren (Min.: 0,5; Max.: 30). Die tägliche Betreuung betrug im Durchschnitt 6,2 h (Min.: 0,5; Max.: 24). Die pflegebedürftigen Personen waren körperlich (z. B. Multiple Sklerose), psychisch (z. B. Depression) oder kognitiv (z. B. Demenz) eingeschränkt.

Die meisten PA nutzten informelle und formelle Hilfen; zwei pflegende Ehemänner und 2 pflegende Söhne beanspruchten keine Hilfe. Der Pflegedienst (*n* = 5) und die Tagespflege/Werkstatt für Menschen mit Behinderung (*n* = 5) wurden am häufigsten genutzt, eine im Haushalt lebende Betreuungskraft (*n* = 2) und betreutes Wohnen (*n* = 1) selten. Informelle Hilfe erhielten PA durch Familie (*n* = 13), Freunde (*n* = 4) und Nachbarschaft (*n* = 5) (Tab. 1).

**Tab. 1:** Beschreibende Merkmale der befragten pflegenden Angehörigen (*n* = 24)

Merkmal	Weibliche Pflegepersonen (*n* = 14)	Männliche Pflegepersonen (*n* = 10)
Alter	61 Jahre (32 bis 77 Jahre)	65,6 Jahre (47 bis 81 Jahre)
Tägliche Pflegezeit	7,2 h (0,5–24 h)	3,7 h (1–8,5 h)
Pflegedauer	6,3 Jahre (1 bis 30 Jahre)	5 Jahre (0,5 bis 17 Jahre)
Beziehung zur pflegebedürftigen Person	7 Lebenspartner	5 Lebenspartnerinnen
	6 Eltern	4 Eltern
	1 Kind	1 Kind
Erkrankung der pflegebedürftigen Person	5 Körperlich	4 Körperlich
	0 Psychosomatisch	1 Psychosomatisch
	9 Kognitiv	5 Kognitiv
Pflegegrad der pflegebedürftigen Person	1 Pflegegrad 1	2 Pflegegrad 1
	6 Pflegegrad 2	4 Pflegegrad 2
	4 Pflegegrad 3	2 Pflegegrad 3
	2 Pflegegrad 5	2 Pflegegrad 4
	1 Pflegegrad 5	0 Pflegegrad 5
Arbeitszeit	3 Vollzeit	5 Vollzeit
	2 Teilzeit	0 Teilzeit
	3 Erwerbslosigkeit	0 Erwerbslosigkeit
	6 Rente	5 Rente
Inanspruchnahme von Hilfe	8 Formelle Hilfe	4 Formelle Hilfe
	11 Informelle Hilfe	5 Informelle Hilfe

### Psychosoziospirituelle Gründe, um Hilfe abzulehnen und anzunehmen

**Abb. 1:**
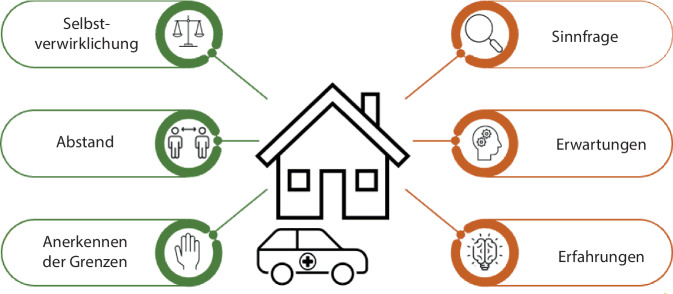
Visuelle Darstellung der Ergebnisse (*grün*: psychosoziospirituelle Gründe für die Inanspruchnahme von Hilfe; *rot*: psychosoziospirituelle Gründe für die Ablehnung von Hilfe)

Es wurden mehrere psychosoziospirituelle Faktoren identifiziert, die beeinflussen, ob Hilfe angenommen oder abgelehnt wird (Abb. 1). Die Kategorien werden mithilfe von einzelnen Zitaten belegt; weitere sind im Anhang aufgeführt (Z1–12).

### Die Sinnfrage

Durch die Pflegebedürftigkeit des Familienmitglieds verändern sich die bisherigen Lebensumstände der PA; der Alltag und die Lebensentwürfe werden nicht selten umgestellt, wodurch sich existenzielle Fragen für die PA ergeben, wie z. B. wozu ist mein Familienmitglied erkrankt, oder wozu übernehme ich die Pflege? Das Beantworten solcher Fragen ist für PA relevant, um einen Sinn zu erfahren. PA erfahren diesen Sinn, indem sie auch in schwierigen Zeiten für ihr Familienmitglied da sein und etwas zurückgeben können und durch ihre Pflege das Wohlbefinden des Familienmitglieds positiv beeinflussen können. Sobald die Pflege durch eine andere Person übernommen wird, greifen die Antworten nur noch bedingt, und die Sinnfrage bleibt zunächst unbeantwortet (Z1–2).

„des ist halt des, was des Leben jetzt von mir will, also es würde sich komplett falsch anfühlen; äh, so lang es noch so geht, irgendeine andere Lösung zu finden. (…) geben und nehmen.“ (Pflegende Schwiegertochter seit 10 Jahren, 51 Jahre alt)

### Die Erwartungen

Die PA nehmen an, dass die Pflege von ihnen erwartet wird (Z3). Sie verspüren Druck und erklären ihn damit, dass 1) sie es der pflegebedürftigen Person versprochen haben, 2) ihr Umfeld ihnen bei der Pflege nicht helfen kann, oder 3) sie andere nicht belästigen wollen. Auch die eigenen Erwartungen zum Alter werden erwähnt; einige PA wollen später ebenfalls in der häuslichen Umgebung versorgt werden (Z4).

„Der Sohn geht no in die Arbeit. (…) i weiß doch selber, was früher war, wo ich fünf Tage in der Woche in da Arbeit ganga bin (…), solangs mir, sog i mal, so einigermaßen geht, will i den ned belästigen.“ (Pflegender Ehemann seit 4 Jahren, 81 Jahre alt)

### Die Erfahrungen

Die meisten PA haben bereits Hilfe beansprucht und waren unzufrieden. Am häufigsten wird formelle Unterstützung kritisiert (Z5–6). Informelle Hilfe enttäuscht, wenn sich Familie und Freunde zurückziehen oder mit Ratschlägen bevormunden; das betrifft besonders pflegende Frauen.

„ein Geschwister kümmert sich und hängt total drin, und die anderen kritisieren und mäkeln und arbeiten dagegen.“ (Pflegende Tochter seit 4 Jahren, 62 Jahre alt)

### Die Selbstverwirklichung

Um Hobbys auszuleben und die notwendigen Freiräume dafür zu bekommen, nutzen PA Hilfsangebote. Jüngere PA erkennen die Relevanz bereits zu Beginn der Pflege; ältere PA bemerken mit zunehmendem Pflegeaufwand, dass sie ihre Bedürfnisse nicht dauerhaft einschränken können (Z7–8).

„des ist das Wichtige. dass man trotzdem noch eben seine Hobbys hat und sich selber noch verwirklichen kann, wenn man nur noch Pflege (…), des schafft man net lang.“ (Pflegende Mutter seit 13 Jahren, 56 Jahre)

### Der Abstand

Das Leben mit einer pflegebedürftigen Person beschreibt eine 68-jährige Ehefrau als „ein miteinander krank sein.“ PA erklären, dass sich die Stimmung der pflegebedürftigen Person auf sie überträgt und sie Zeit ohne die Person verbringen wollen; dies trifft besonders auf PA von kognitiv und psychisch erkrankten Personen zu. Pflegende Kinder wahren sich ihre interpersonalen Grenzen v. a. durch getrennte Wohnräume; pflegende Lebenspartner/-innen und Eltern verlassen eher die Pflegesituation (Z9–10).

„i brauch aber, wie gsagt, meine Auszeiten, und die brauch i scho, die oan oder andere Stund (…), wenn i mi = m Hund unterwegs bin (…), wird der Kopf schee frei.“ (Pflegender Ehemann seit 1,5 Jahren, 72 Jahre alt)

### Das Anerkennen der Grenzen

Einige PA entscheiden bewusst, bei welchen Tätigkeiten sie Hilfe benötigen. Eine 75-jährige Ehefrau lehnt die Körperpflege ihres Mannes ab; „mich würds ekeln; er is mein Mann und früher hatten wir auch a Sexleben (…) da würd ich mich abgrenzen.“ Häufig nehmen PA ihre Grenzen erst im Verlauf der Pflege wahr. Ältere PA können mit steigendem Pflegebedarf körperlich anspruchsvolle Tätigkeiten nicht mehr durchführen. Bei Partnerschaften fällt auf, dass es häufig eine klare Aufgabenteilung vor der Pflegebedürftigkeit gab, so wurde der Haushalt von den Frauen, bürokratische Angelegenheiten wurden von den Männern übernommen. Mit Beginn der Pflege versuchten die PA, die Tätigkeiten mitzuübernehmen, suchten nach einiger Zeit jedoch konkrete Hilfe (Z11–12).

## Diskussion

In der Studie mit insgesamt 24 PA beanspruchten 20 Personen formelle und/oder informelle Hilfe, und 4 nutzen keine Hilfe. Es zeigte sich, dass PA Hilfe nur dann (erneut) beanspruchen, wenn sie als wertvoll wahrgenommen wird. Diese Wahrnehmung wird jedoch nicht nur von der Qualität der Hilfe beeinflusst, sondern vielmehr davon, ob PA den Wert für die eigene Person erkennen; sie sollten einen Sinn darin erfahren, Hilfe anzunehmen. In der Erhebung wurde jedoch ein Problem deutlich; häufig erfahren PA keinen Sinn, wenn sie Hilfe beanspruchen, da sie die Pflege ihres Familienmitglieds damit begründen, dass sie für die Person da sein können und sich ihre Pflege positiv auf das Wohlbefinden der pflegebedürftigen Person auswirkt. PA erfahren die Pflege als ihre Lebensaufgabe und lehnen Hilfe bewusst ab, um diesen Sinn weiterhin zu spüren.

Zwei Reviews zu Spiritualität bei PA bestätigen das Ergebnis; PA, die den Sinn in der Erkrankung und Pflegebedürftigkeit ihres Familienmitglieds darin erfahren, dass es ihre Bestimmung ist, für die Person da zu sein, schaffen es seltener, eigene Bedürfnisse wahrzunehmen, Grenzen zu erkennen und Hilfe zuzulassen. Die Studien zeigen aber auch, dass PA den Sinn ebenfalls in anderen Lebensinnhalten finden können, z. B., dass sie sich durch die Pflege weiterentwickeln oder die eigene Gesundheit bewusster wahrnehmen. Diese Inhalte wirken sich positiv auf eine achtsame Lebenseinstellung aus, wodurch Hilfe leichter zugelassen werden kann [8, 9].

Bereits Haußmann stellte in ihrer Studie zu Religiosität und Spiritualität bei PA fest, dass das Erfahren von Sinn ein spirituelles Grundbedürfnis für PA ist, welches Orientierung und Halt geben kann [12]. Wenn die Pflege als Aufgabe oder als alleiniger Lebenssinn verstanden wird, kann Hilfe zu Orientierungslosigkeit führen und als bedrohliche Infragestellung wahrgenommen werden.

Bereits in vorherigen Studien gaben PA an, dass sie auf Hilfe verzichten, weil sie die Pflege als Aufgabe empfinden, sie die Verantwortung selbst tragen wollen und die Hilfe ihrem Anspruch nicht gerecht wird [4, 5], auch in dieser Studie wurden negative Erfahrungen mit Hilfe gemacht. Formelle Hilfe steht zwar nicht grundlos in der Kritik [4–6], jedoch ist es durch Spiritualität möglich, die eigene Lebenssituation gesamthaft zu reflektieren und sich auf die positiven Aspekte der Hilfe zu fokussieren. Die möglichen Qualitätseinbußen können dann als Kompromiss verstanden werden, damit häusliche Pflege weiter gewährleistet werden kann.

Ein weiterer hinderlicher Grund ist, dass PA den Druck verspüren, dass ihr Umfeld von ihnen erwartet, dass sie die Pflege übernehmen. Eine Studie zu geschlechtsspezifischen Besonderheiten der häuslichen Pflege bestätigt, dass sich v. a. Frauen in die Rolle der Pflegeperson gedrängt fühlen. Da sie in der Rolle nicht versagen wollen, schämen sie sich, wenn sie Hilfe benötigen [6]. Die VDK-Studie zeigt jedoch, dass Familie und Freunde die Hauptpflegeperson meistens unterstützen wollen [3].

Die Studie zeigt zudem, dass PA mit Hinblick auf das eigene Älterwerden, ihrer Familie vorzuleben versuchen, wie sie später gepflegt werden möchten und Hilfe eher ablehnen.

Ob Hilfe angenommen wird oder nicht, hängt nicht nur von der Qualität ab, sondern davon, ob PA einen Sinn in der Pflegesituation erfahren und bereit sind, sich von Erwartungen zu lösen und sich flexibel neuen Gegebenheiten anzupassen. In der Pflegeberatung nach SGB XI sollte daher zunächst besprochen werden, wozu Hilfe (nicht) angenommen wird, und erst im zweiten Schritt sollte die Hilfe vorgestellt werden. Bacher schlägt vor, PA zu unterstützen, ihre Werte und Ziele über die Pflegesituation hinaus zu betrachten, um mögliche Hindernisse wie z. B. Gefühle der Verpflichtung zu identifizieren. Ergänzend hierzu sollten die Selbstentfaltungswerte, die PA helfen, Hemmschwellen zu überwinden und Hilfe zuzulassen, stehen [18]. In dieser Studie werden neben der Selbstverwirklichung noch Abstand und Grenzen als psychosoziospirituelle Faktoren erkannt, die positiv auf die Inanspruchnahme von Hilfe wirken. Die begünstigenden Faktoren sollten in der Pflegeberatung gezielt thematisiert werden. Fragen wie „Welche Lebensinhalte tragen mich über die Pflege hinaus und geben mir Energie?“ oder „Was möchte ich in der Betreuung übernehmen, was nicht?“ sollten in der Pflegeberatung angesprochen werden, damit PA ihre Rolle als Pflegeperson gesamthaft reflektieren, mit den Benefits und den Herausforderungen. Eine gezielte psychosoziospirituelle Beratung kann zu Beginn der Pflege deutlich nachhaltiger sein als eine Leistungsberatung. Um diesem Bedarf gerecht zu werden, benötigt es langfristig eine entsprechende Qualifizierung der Beratenden.

### Limitation

Mit dem Prinzip der maximalen Variationsbreite wurden ausreichend Daten gesammelt, sodass sich bei der Analyse keine neuen Kategorien mehr zeigten. Es ist anzunehmen, dass die Ergebnisse auch auf PA aus anderen Bundesländern sowie PA, welche keinen Pflegegrad für die zu pflegende Person beantragt haben, übertragen werden können. Eine Überprüfung ist dennoch zu empfehlen.

Die Angaben zu Pflegedauer, täglicher Pflegezeit und Inanspruchnahme von Hilfe sind Selbsteinschätzungen der PA und müssen nicht den objektiven Werten entsprechen.

Die meisten Teilnehmenden wurden mithilfe des MDK rekrutiert. Das Abhängigkeitsverhältnis zwischen PA und MDK und der Bias der sozialen Erwünschtheit sind kritisch zu reflektieren.

Die Ergebnisse wurden nicht an die Teilnehmenden rückgekoppelt, da es sich um eine Längsschnittstudie handelt und die Auswertung der zweiten Erhebung noch aussteht.

## Ausblick

Die Studie bestätigt, dass PA nicht selten mit inneren Konflikten konfrontiert sind, wenn sie entscheiden, ob sie sich in der häuslichen Pflegesituation unterstützen lassen. Zum einem wollen sie ihre eigenen Bedürfnisse und Grenzen wahren, zum anderen wollen sie den eigenen, familiären und gesellschaftlichen Erwartungen gerecht werden. Es erscheint sinnvoll, PA zunächst professionell bei der Auflösung des inneren Konflikts zu begleiten, bevor im Rahmen der gesetzlich geregelten Pflegeberatung Hilfsangebote vorgestellt werden. Eine wissenschaftliche Begleitung des Ansatzes ist empfehlenswert.

## Fazit für die Praxis

- Pflegende Angehörige lehnen Hilfe nicht nur aufgrund der Qualität des Angebots ab, sondern vielmehr, weil sie diese nicht mit den eigenen, familiären und gesellschaftlichen Werten und Erwartungen vereinbaren können.
- Pflegende Angehörige profitieren davon, wenn sie professionell dabei begleitet werden, die eigenen, familiären und gesellschaftlichen Werte und Erwartungen zu reflektieren. Damit können Barrieren identifiziert und Hemmschwellen in Bezug auf Hilfsangebote abgebaut werden.

## Supplementary Information

Anhang

## Data Availability

Die erhobenen Datensätze können auf begründete Anfrage in anonymisierter Form beim korrespondierenden Autor angefordert werden.
