# Supplementary material for: Between experiencing meaning and self-realization: a qualitative study on utilization of help from the perspective of family caregivers
Source: Z Gerontol Geriatr. 2025 Apr 8;58(7):583–9. [Article in German] doi: 10.1007/s00391-025-02437-1 (PMC12568785; doi:10.1007/s00391-025-02437-1)
Supplement: Supplementary file 1 — Anhang [file 391_2025_2437_MOESM1_ESM.docx]

Anhang

1. Guideline for qualitative research

| No | | Item | | Guide questions and answers |
| --- | --- | --- | --- | --- |
| **Domain 1: Research team and reflexivity** | | | | |
| 1 | | Interviewer/facilitator | | Which author/s conducted the interview or focus group?  *Jenny Kubitza, Verena Steinmaier, Hannah Kesselmayer* |
| 2 | | Credentials | | What were the researcher’s credentials?  *Jenny Kubitza M.Sc, Verena Steinmaier B.Sc., Hannah Kesselmayer B.Sc., Ruth Mächler PhD, Dagmar Teutsch M.A., Eckhard Frick Prof. PhD* |
| 3 | | Occupation | | What was their occupation at the time of the study?  *Jenny Kubitza, Ruth Mächler and Dagmar Teutsch have been senior researchers in the Department of Spiritual Care.  Verena Steinmaier and Hannah Kesselmayer are employed as student assistants in the qualitative project and are closely supervised by Jenny Kubitza. Eckhard Frick has the professorship for Spiritual Care. He also works as a psychotherapist in his own praxis.* |
| 4 | | Gender | | Was the researcher male or female?  *Five of the researchers are female, one researcher is male.* |
| 5 | | Experience and training | | What experience or training did the researcher have?  *All researchers are familiar with qualitative social research and, except for Hannah Kesselmayer, have already conducted other qualitative projects.* |
| 6 | | Relationship established | | Was a relationship established prior to study commencement?  *Prior to the interview, the interviewer and participant met by phone to provide information about the study and to review inclusion and exclusion criteria.* |
| 7 | | Participant knowledge of the interviewer | | What did the participants know about the researcher?  *Participants received an information sheet prior to the interview that provided information about the reasons, aims, and study design. In addition, they were told the name, professional degree, and the current occupation of the interviewer.* |
| 8 | | Interviewer characteristics | | What characteristics were reported about the interviewer/ facilitator?  *They were told the name, gender, professional degree, and the current occupation of the interviewer.* |
| **Domain 2: study design** | | | | |
| 9 | Methodological orientation and Theory | | What methodological orientation was stated to underpin the study?  *Deductive and inductive content analysis according to Mayring* | |
| 10 | Sampling | | How were participants selected?  *Participants were recruited in two ways: (1) via flyers that have been distributed personally to family caregivers at self-help groups and uploaded on social networks for family caregivers; and (2) after participation in the quantitative study, flyers were sent by post to family caregivers.* | |
| 11 | Method of approach | | How were participants approached? *The participants contacted the research team by mail or telephone.* | |
| 12 | Sample size | | How many participants were in the study?  *24 participants* | |
| 13 | Non-participant | | How many people refused to participate or dropped out? Reasons?  *Two people were no longer interested to participate in the study after telephone information was provided. No person dropped out of the interview. There were other requests for interviews, but they were declined by the research team due to data saturation.* | |
| 14 | Setting of data collection | | Where was the data collected?  *Interviews were conducted in participants' own homes in person, by telephone, or by videoconference. Some interviews were conducted in the offices of the research team based on the request of some participants.* | |
| 15 | Presence of non-participants | | Was anyone else present besides the participants and researchers?  *As the interviews took place in the participants' homes, family members were present in the background. In some cases, the person being cared for needed help from the participants, so the interview had to be paused.* | |
| 16 | Description of sample | | What are the important characteristics of the sample? *14 women and 10 men were interviewed. All participants care for a relative in a home setting in Bavaria, Germany. They have been caring for the persons for an average of 5,4 years (min.- max.: 0,5 – 30 years). These are spouses, parents, or children who need physical, psychological, or cognitive help. Participants range in age from 32 to 81 years old, whereby the average age of the women (61) is slightly younger than the average age of the men (65).* | |
| 17 | Interview guide | | Were questions, prompts, guides provided by the authors? Was it pilot tested?  *A guide was developed within the research team that included a narrative stimulus as well as follow-up questions. The guide was flexible to the interviews. The first two interviews were used to test the guide. As the interviews resulted in relevant data, they were included in the analysis.* | |
| 18 | Repeat interviews | | Were repeat interviews carried out? If yes, how many?  *No.* | |
| 18 | Audio/visual recording | | Did the research use audio or visual recording to collect the data?  *Audio.* | |
| 20 | Field notes | | Were field notes made during and/or after the interview or focus group?  *No.* | |
| 21 | Duration | | What was the duration of the interviews or focus group?  *The 24 interviews had an average duration of 45.5 minutes, with a minimum of 27.61 minutes and a maximum of 1.31.16 hours. The interviews with men took slightly longer at 51.6 minutes than with women at 41.15 minutes* | |
| 22 | Data saturation | | Was data saturation discussed?  *Yes.* | |
| 23 | Transcripts returned | | Were transcripts returned to participants for comment and/or correction?  *No, this aspect is also discussed in the limitations.* | |
| **Domain 3: anaylsis and findingsz** | | | | |
| 24 | Number of data codes | | How many data coders coded the data?  *Jenny Kubitza coded most of the data. Verena Steinmaier coded three interviews. The codes were discussed and modified throughout the research team.* | |
| 25 | Description of the coding tree | | Did authors provide a description of the coding tree?  *The results describe the part of the coding tree relevant to the research questions of the article.* | |
| 26 | Derivation of themes | | Were themes identified in advance or derived from the data?  *Jenny Kubitza and Verena Steinmaier coded inductively and deductively.* | |
| 27 | Software | | What software, if applicable, was used to manage the data?  *MAXQDA 22 Software* | |
| 28 | Participant checking | | Did participants provide feedback on the findings?  *A part of the results has been given back to some participants who agreed to be involved in the process of evaluation.* | |
| 29 | Quotations presented | | Were participant quotations presented to illustrate the themes / findings? Was each quotation identified?  *Yes.* | |
| 30 | Data and findings consistent | | Was there consistency between the data presented and the findings?  *Yes.* | |
| 31 | Clarity of major themes | | Were major themes clearly presented in the findings?  *Yes.* | |
| 32 | Clarity of minor themes | | Is there a description of diverse cases or discussion of minor themes?  *Different cases are discussed for their similarities and differences with regard to the research questions.* | |

1. Repräsentative Zitate

| **Tabelle: Repräsentative Zitate für die Themen** | | |
| --- | --- | --- |
| Nr. | Subthemen | Zitate |
| **Thema 1: Gründe, um Hilfe abzulehnen** | | |
| 1  2 | Die Sinnfrage | *„wenn ich mir dann noch vorstelle, ich würde meine Frau in ein Pflegeheim geben außerhalb des Hauses; (…) dann wüsst ich überhaupt nicht mehr was ich für=n Ansatz empfinden müsse (…) wo ist da die Aufgabe für mich, wo?“* (Pflegender Ehemann seit 11 Jahren, 72 Jahre alt)  „*es ist für mich einfach äh selbstverständlich, dass man des macht; und äh ich mach=s auch gerne, wenn äh weil es was ist, was in meiner Macht steht.“*  (Pflegende Mutter seit 13 Jahren, 56 Jahre alt) |
| 3  4 | Erwartungen | *„ich hab zwei ältere Brüder, die sich aber halt nicht kümmern. und mich vorsichtshalber auch gar nicht anrufen, weil am Ende würd ich ja sagen, du könntest auch mal eine Aufgabe übernehmen.“*  (Pflegende Tochter seit 2 Jahren, 52 Jahre alt)  *„wenn solche Sachen thematisiert werden, dann sind meine Kinder ja auch dabei. ich bemüh mich, (…) denn irgendwann bin ja vielleicht ich es, der ein Pflegefall ist.“*  (Pflegender Sohn seit 1 Jahr, 47 Jahre alt) |
| 5  6 | Erfahrungen | „*dann hab ich meinen Mann vier Monate im Pflegeheim gehabt.(...) mein Mann ist untergegangen. der ist verzweifelt gewesen. hat mir auch nicht gut getan. weil ich gesehen hab, wie der unterversorgt ist und wie er leidet drunter.“* (Pflegende Ehefrau seit 30 Jahren, 67 Jahre alt)  *„als ich im Krankenhaus lag (…), hat meine Tochter dann einen ambulanten Pflegedienst organisiert. (…) die wussten ja teilweise gar nicht, was sie machen sollten. und dann stehen die ja derartig unter Zeitdruck (…), dass sich die ja (…) um die Psyche äh der Erkrankten oder der Pflegebedürftigen an und für sich gar net kümmern können;“*  (Pflegender Ehemann seit 11 Jahren, 73 Jahre alt) |
| **Thema 2: Gründe, um Hilfe anzunehmen** | | |
| 7  8 | Die Selbst-verwirklichung | *„ich möchte für mich dies Wochenende möglichst frei haben (…) weil (…) man kann sich ja überlegen, dass ich am Wochenende gehe, weil sie da eben nicht in der Tagespflege ist, aber dann ist mein Wochenende eben auch zerschossen.“* (Pflegende Tochter seit 2 Jahren, 52 Jahre alt)  „*ich find man muss halt dann immer des Beste draus machen. aber was ich jetzt zum Beispiel vor hab, Februar oder März 14 Tag zu verreisen. (…) hab ich ihr auch scho g=sagt. sie hat g=sagt, (..) hab scho recht(...) und des hab ich halt so vor. entweder Türkei oder Ägypten (…) hau ich 14 Tage ab, ja. (…) also dass ich mir was Gutes tu. (…). ich finde des hab ich dann auch verdient.“*  (Pflegender Vater seit 2 Jahren, 79 Jahre alt) |
| 9  10 | Abstand | *„ich setz mich auch mal raus, vor die Tür. da hab ich a Bank und da lass ich einfach mal zehn Minuten gute Zeit lassen, ja?“*  (Pflegende Ehefrau seit 3 Jahren, 68 Jahre alt)  d  *„ich weiß gar nicht, das war im Frühling irgendwann, wo ich=s festgestellt hab, dass er [das Essen] vergisst; dass ich dann mittags immer angerufen hab, also auch an dem Tag, wo ich nicht da war, und dies hat jetzt meine Schwester übernommen. sodass ich an dem Tag, wo ich nicht bei ihm bin, sozusagen mal den ganzen Tag äh frei hab; und nicht immer an irgendwas denken muss.“*  (Pflegende Tochter seit 2 Jahren, 66 Jahre alt) |
| 11  12 | Anerkennen der Grenzen | *„wenn ich wirklich ganz präzise jemanden bitte, kannst du mir da oder da mal helfen; weil ich da selber einfach zu ungeschickt oder zu wenig Kraft oder was immer; also man erfährt sehr viel; (…) und ich frag natürlich schon gezielt eine Person; von der ich annehm der oder die kann das.“*  (Pflegende Ehefrau seit 5 Jahren, 81 Jahre alt)  *„also körperliche Pflege (…) geht los langsam würde ich sagen. (…) und das ist für uns dann schon, wäre eine Überwindung glaub ich, da mitzuhelfen. also waschen und so weiter, da würd ich schon, vom Gefühl her sagen das wäre besser wenn das jemand von der Pflegestation macht. (…) ich hab auch Zivildienst gemacht, (…) aber die eigene Mutter da körperlich zu pflegen ist glaub ich schon bisschen schwieriger, ne?*  (Pflegender Sohn seit 1 Jahr, 53 Jahre alt) |
